# Patient-Specific Computational Models of Coronary Arteries Using Monoplane X-Ray Angiograms

**DOI:** 10.1155/2016/2695962

**Published:** 2016-06-15

**Authors:** Ali Zifan, Panos Liatsis

**Affiliations:** ^1^School of Medicine, University of California, San Diego, CA 92093, USA; ^2^Department of Electrical Engineering, The Petroleum Institute, P.O. Box 2533, Abu Dhabi, UAE

## Abstract

Coronary artery disease (CAD) is the most common type of heart disease in western countries. Early detection and diagnosis of CAD is quintessential to preventing mortality and subsequent complications. We believe hemodynamic data derived from patient-specific computational models could facilitate more accurate prediction of the risk of atherosclerosis. We introduce a semiautomated method to build 3D patient-specific coronary vessel models from 2D monoplane angiogram images. The main contribution of the method is a robust segmentation approach using dynamic programming combined with iterative 3D reconstruction to build 3D mesh models of the coronary vessels. Results indicate the accuracy and robustness of the proposed pipeline. In conclusion, patient-specific modelling of coronary vessels is of vital importance for developing accurate computational flow models and studying the hemodynamic effects of the presence of plaques on the arterial walls, resulting in lumen stenoses, as well as variations in the angulations of the coronary arteries.

## 1. Introduction 

Coronary heart disease (CHD), also called coronary artery disease (CAD), is globally the leading cause of death and is predicted to remain so for the next 20 years. In 2020, it is estimated that this disease will be responsible for a total of 11.1 million deaths globally [1]. In Europe, between 1 in 5 and 1 in 7 European women die from CAD, and the disease accounts for between 16% and 25% of all deaths in European men [2]. According to American Heart Association (AHA) statistics, coronary heart disease alone caused nearly 1 of every 7 deaths in the United States in 2011. In 2011, 375,295 Americans died of coronary heart disease. Each year, an estimated 635,000 Americans have a new coronary attack (defined as first hospitalized myocardial infarction or coronary heart disease death) and nearly 300,000 have a recurrent attack [3]. In addition to its mortality burden, CAD is a leading cause of morbidity and loss of quality of life. This makes CAD a major public health problem, which exerts heavy economic costs.

The most common cause of CAD is atherosclerosis, which is caused by the presence of plaques growing in the coronary arteries until the blood flow to the heart's muscle is limited, resulting in lumen stenosis. If the clot becomes large enough, it can mostly or completely block the flow of oxygen-rich blood to the part of the heart muscle fed by the artery. This can lead to angina, myocardial infarction, or necrosis [4]. Therefore, early detection and diagnosis of CAD is particularly important for reduction of the mortality and subsequent complications. The distribution of plaques within the coronary artery is not homogenous due to local conditions that induce plaque formation and progression [5, 6]. Plaque formation is commonly found in areas of low shear stress or regions of turbulent flow, for example, left coronary bifurcation [7].

Computational fluid dynamics (CFD) allows for efficient and accurate computations of hemodynamic features of both normal and abnormal situations in the cardiovascular system and in vivo simulations of coronary artery flow changes [8, 9]. It also allows for the study of hemodynamic changes of the coronary artery, even before the plaques are actually formed in the coronary artery wall or the development of vessel occlusion. The current work aims at developing patient-specific 3D computational models of the arterial tree from anatomical information provided by 2D monoplane coronary angiograms, thus facilitating the investigation of hemodynamic characteristics in simulated coronary models, thus contributing to the identification of patients with potential risks of developing coronary artery disease.

In certain circumstances, treatment of coronary disease can be achieved without surgery. Angioplasty is a nonsurgical procedure, which is often used to open blocked coronary arteries. Despite the high resolution images obtained in cardiovascular imaging using powerful imaging techniques, such as Multislice Computed Tomography (CT) [10, 11], Electron-Beam CT [12, 13], and Magnetic Resonance Imaging (MRI) [14, 15], conventional coronary angiography remains the “gold standard” for the assessment of coronary artery disease [16]. In this procedure, first, cardiac catheterization is performed, where a sheath is inserted into an artery. Next, a catheter is passed through the sheath and guided up the blood vessel to the arteries surrounding the heart chambers. A small amount of contrast material is injected through the catheter and is photographed as it moves through the heart's chambers, valves, and major vessels. From the resulting 2D X-ray images of the contrast material, the surgeon can tell whether the coronary arteries are narrowed and/or whether the heart valves are working correctly.

The 2D nature of the images further complicates the process, as it is, in general, difficult to assess overlapping and parallel vessels. Furthermore, other important anatomical characteristics of the arterial tree such as vessel curvature, torsion, and bifurcation take-off angles may not be reliably assessed using only 2D angiographic images. As discussed previously, these latter parameters may be important for the study of hemodynamic factors related to atherosclerosis [9, 17]. Therefore, a realistic model of the coronary tree can alleviate the aforementioned complications, by first providing a 3D view of the vessels, which makes it significantly easier for the physician to detect stenoses or aneurisms. Secondly, the model can be used for further CFD analysis.

There have been a number of studies in the literature for the quantitative determination of the 3D representation of the coronary tree based on angiographic views [18–32]. In the former approaches, two or more projection images from different viewing directions are used to reconstruct the vessel centrelines in three dimensions. Our research team has also contributed to the development of 3D coronary vessel models from monoplane angiogram images [33, 34] and experimented with fluid flow simulations in the resulting mesh models of the vessels [35].

In this research, we introduce a semiautomatic method for reconstruction of coronary vessels in 3D from two monoplane angiogram images. The proposed approach incorporates a new robust edge extraction algorithm, where the user can choose any pair of start and end points along a vessel in order to obtain its 3D reconstructed volume, using the iterative 3D reconstruction approach, proposed previously by our group [33]. Following the selection of the points by the user, the process is fully automatic and does not require any user intervention.

## 2. Materials and Methods

We present a methodology for reconstructing realistic topologies from arterial trees using accurate vessel wall positions from two conventional monoplane angiograms. The proposed method involves four main steps: (a) automatic centreline extraction, (b) a novel automatic edge detection method, (c) an iterative method for 3D centreline reconstruction, which results in an accurate representation of the main components of the arterial tree, namely, Left Anterior Descending (LAD) or Left Circumflex Artery (LCX), and finally (d) vessel surface reconstruction using vessel diameter information and intrinsic coordinates. The end result is a 3D surface representation of the arterial tree, which can subsequently be meshed using suitable Finite Element mesh generation software and used in computational fluid dynamics (CFD) simulations for hemodynamic assessment in cardiology applications.

### 2.1. X-Ray Acquisition

The images were acquired using a Philips Integris 3000H X-Ray C-arm unit with an under couch tube/over couch image intensifier configuration. The projections obtained during routine coronary intervention of 5 stenotic patients using pulsed fluoroscopy (12.5 p/s) were LAO, 30° LAO caudal, 30° LAO cranial, anteroposterior with cranial and caudal angulations, RAO, 30° RAO caudal, and 30° RAO cranial and left lateral. As the image acquisition process is ECG-gated, the phase of the heart cycle for each frame can be determined. Other gantry information, such as the focal spot to image intensifier distance (SID), field of view (FOV), and gantry orientation, was automatically recorded and stored with each image file and included in DICOM 3.0 image format. All procedures were performed in accordance with institutional guidelines, and all patients gave informed consent before PCI.

### 2.2. 3D Centreline Reconstruction

The multistage 3D centreline reconstruction procedure consists of the following steps: (1) vessel enhancement, (2) hysteresis thresholding, (3) skeletonization, (4) bifurcation and end point detection, and finally (5) 3D centreline reconstruction. We use an automatic approach similar to the one proposed by the authors [34] in order to extract a skeletonized representation of the arterial tree from projected 2D images.

**Figure 1:**
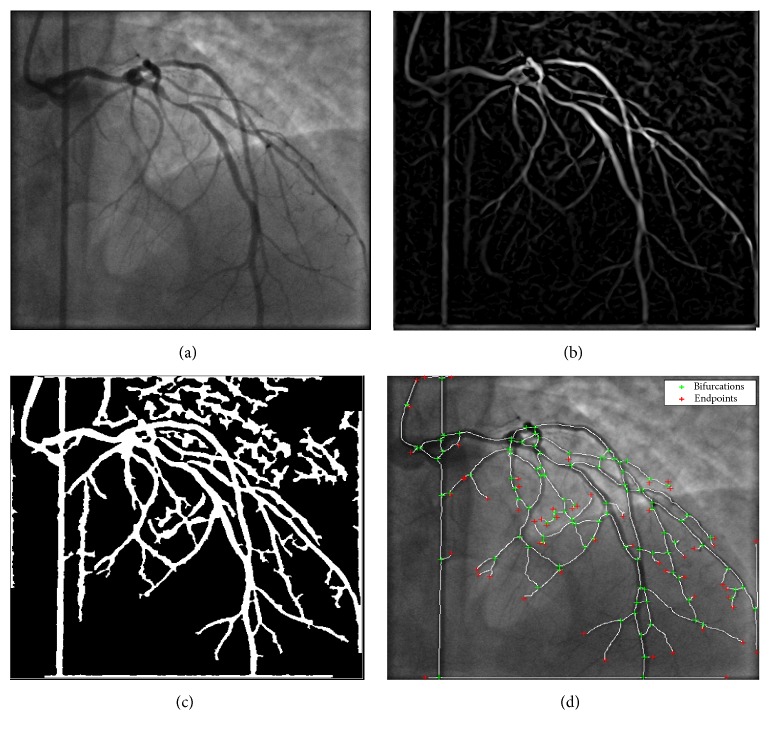
Automatic coronary vessel skeletonization: (a) angiogram image, (b) vessel enhanced model, (c) binarized arterial tree, and (d) skeletonization followed by bifurcation and end point detection for arterial tree branch labelling.

**Figure 2:**
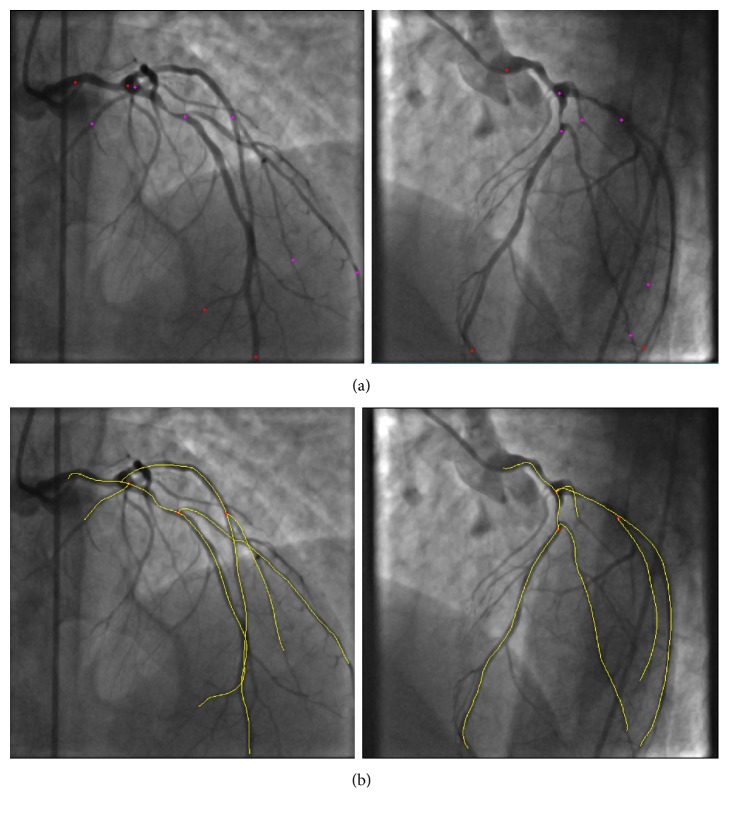
Vessel centreline selection: (a) the user selects points on the arteries on both projections; (b) the corresponding centreline segments are extracted and are spline-interpolated to yield the final centrelines.

The algorithm uses a multiscale vessel enhancement method based on the eigenvalues of the Hessian matrix of the angiogram images in order to enhance the arterial tree and following the application of morphological operations, the final centreline, bifurcation, and end points are automatically extracted from the angiogram image. A sample centreline extraction of a patient angiogram is shown in Figure 1. In the proposed method, the user is only required to select the start and end points of the vessel sections of interest. This is shown in Figure 2(a) for a sample selection on the LAD, LCX, and several branches. The centreline extraction method starts by finding the closest point on the extracted centreline of Figure 1(d) to the start and end points, selected by the user using an interactive tool in the Euclidean distance sense. Next, the segment of the vessel which falls in between the selected points is extracted from the binary centreline image of Figure 1(d). Finally, spline interpolation is carried out to obtain a smooth curve, but this time, the bifurcation points that lie on the path are not used, as they do not fall on the centre position of the vessel at the point of branching. However, their coordinates are stored for later usage in the 3D centreline reconstruction stage. The resulting process can be visualized in Figure 2(b).

Once the centrelines of interest are obtained, a 3D representation of the centreline is generated using the concept of epipolar geometry [33]. This method requires the parameters of the monoplane imaging system for the 3D reconstruction, including the distance between each focal spot and the image plane (source to intensifier distance, SID), the field of view (FOV) in terms of pixel size, the distance between the focal spots and the rotation angle, and translation between the different views taken. Due to the curvature of the arterial branches, foreshortening is eliminated through an iterative process. Since two images are not enough to provide noncylindrical vessel cross-sectional shapes, the reconstruction algorithm assumes a circular vessel cross-sectional area.

**Figure 3:**
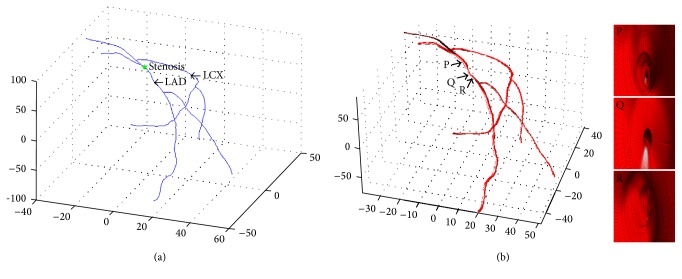
3D vessel reconstruction. (a) Reconstructed 3D vessel centrelines from left and right 2D projections, (b) 3D reconstructed coronary tree alongside an inside view of the reconstructed vessel at three orthogonal planes. The stenosis can be clearly observed in the middle image as a sudden reduction in vessel diameter.

The final reconstructed arterial tree is obtained by connecting the various arterial branches, as shown in Figure 3(a). The latter method is iterative, which minimizes foreshortening effect.

### 2.3. Vessel Edge Extraction

Once the coronary vessel centreline points are reconstructed in 3D, knowledge of the vessel lumen diameter is required in order to construct the 3D vessel lumen surface. The common method is to use information about the catheter size and scale accordingly for the diameter of the vessels. However, foreshortening may affect the vessel's diameter on the projected planes. In what follows, we discuss a novel edge extraction method to address this.

**Figure 4:**
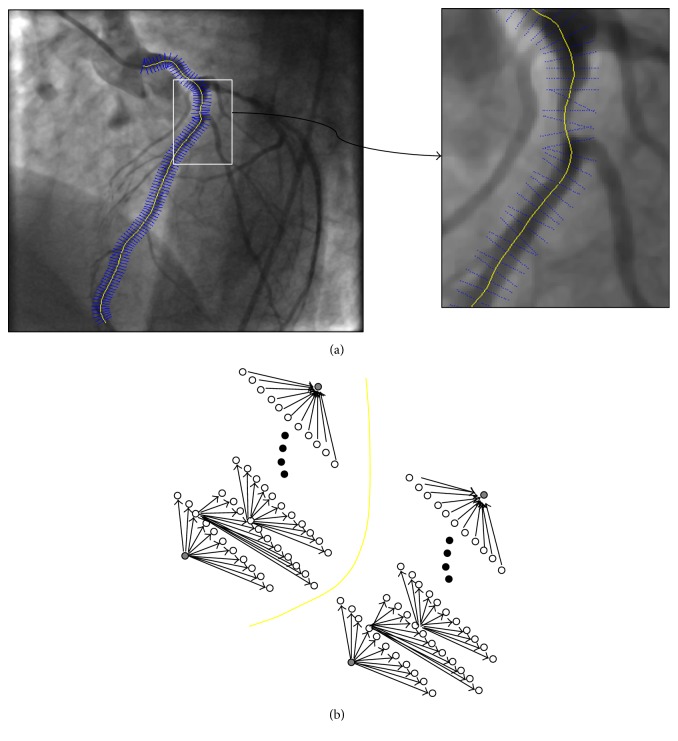
Edge extraction: (a) normals (blue) to the centreline (yellow) are drawn, (b) an edge as a directed graph.

In order to find the vessel walls, starting at the initial centreline point, we build normals to the centreline, comprised of 10 points of equal spacing on each side of the centreline. This ensures that the vessel walls are covered within this range. The latter gridding is depicted in Figure 4(a). Next, we use numerical differentiation in order to calculate the first- and second-order image derivatives at all of these points. Ideally, what we aim for is a smooth curve, which is placed correctly along the boundaries of each of the selected vessels. The smoothness constraint implies that the local edge direction differences should be small from pixel to pixel, and the correct placement of the curve on the vessel walls suggests that each edge pixel should have a relatively large gradient magnitude. A corollary of the latter is that there should be a small difference between successive edge points in terms of the magnitude of the gradient and direction of the edge candidates. In other words, their difference must fall below a certain threshold (i.e., in terms of optimisation approach, this should translate to a lower cost). Moreover, to add more accuracy to the edge detection algorithm, we include a second-order derivative component in our cost function. The basic principle behind this is that the position in an image, where the second-order derivatives become zero, is also an edge candidate. Thus, we add the second-order derivative term as another constraint in our cost function. In other words, we pose the problem of coronary boundary detection as a convex optimization problem by minimizing a cost function, which incorporates both a curvature constraint and a global constraint that a vessel boundary has to satisfy in order for the proposed restricted search method to be applicable.

We approach the problem of edge curve extraction in the angiogram images from a graph theory point of view. In our approach, the generation of a vessel wall curve from a start point *o*_1_ to an end point *o*_*N*_ *is* equivalent to the generation of a minimum-cost path in a directed graph from *o*_1_ to *o*_*N*_, as shown in Figure 4(b). Here, we denote the edge magnitude of a node *o*_*i*_ (where *i* < *N*) with *e*_*gm*_, its second-order derivative magnitude with *e*_*hm*_, and its direction *e*_*gd*_, respectively, and define a cost function using the former constraints on smoothness and edge magnitude. We seek to optimize the following cost function:

(1) Co1,o2,…,oN=∑i=1Nemoi−β∑i=2Negmoiehmoi+ε−egmoi−1ehmoi−1+ε−1−β∑i=2Negdoi−egdoi−12,

where *β* is a hyperparameter, which controls the trade-off between 1st- and 2nd-order edge magnitude and smoothness (i.e., edge direction), and *ε* is a small positive number, which prevents numerical instabilities. Finding a minimum-cost path of the above formula using a brute-force approach is not trivial in terms of computation. Therefore, we use dynamic programming to search for the optimal edge nodes on this graph. In order to do so, we divide the problem recursively into smaller subproblems, which may need to be subsequently solved, solving each subproblem and storing the solutions in a look-up table. In other words, we split the path between the nodes *o*_1_ and *o*_*N*_,* into* two optimal subpaths *o*_1_ *o*_*i*_ and *o*_*i*_ *o*_*N*_ for any *o*_*i*_ lying on the optimal path *o*_1_ *o*_*N*_. The objective function (1) can be written in a recursive form as follows:

(2) $$\begin{array}{c} C\left(\;o_{1},o_{2},\ldots ,o_{i}\;\right)=C\left(\;o_{1},o_{2},\ldots ,o_{i-1}\;\right)+f\left(\;o_{i-1},o_{i}\;\right), \end{array}$$

where

(3) foi−1,oi=emoi−βegmoiehmoi+ε−egmoi−1ehmoi−1+ε−1−βegdoi−egdoi−12.

Then, the complete optimization problem becomes

(4) Co^1,o^2,…,o^i=arg maxOj⁡Co1,o2,…,oij=1,2,…,i,

where ${\hat{o}}_{1},\ldots ,{\hat{o}}_{i}$ are the optimal nodes selected at each level of the graph, chosen as the optimal vessel wall point. The optimal path ${\hat{o}}_{1}{\hat{o}}_{i}$ itself can be split into two optimal subpaths ${\hat{o}}_{1}{\hat{o}}_{i-1}$ and ${\hat{o}}_{i-1}{\hat{o}}_{i}$, which satisfy the following recursive relation:

(5) Co^1,o^2,…,o^i=arg maxOiCo^1,o^2,…,o^i−1+fo^i−1,oi,

where

(6) $$\begin{array}{c} C\left(\;{\hat{o}}_{1}\;\right)=e_{m}\left(\;o_{1}\;\right). \end{array}$$

Thus we reduced the optimization of *N* stages to a two-variable optimization. As we need single nodes for the initial start and end nodes, the former are chosen as the nodes on the normals having the maximum gradient magnitude. One of the advantages of the proposed vessel wall extraction method is that it is a sequential edge-oriented approach rather than a region-oriented one, which takes into account the spatial context of the vessels, as opposed to a pixel-by-pixel segmentation that would not be possible in the presence of noise and loss of coherence in the vessel structures.

### 2.4. Three-Dimensional Vessel Wall Reconstruction

The vessel surface reconstruction is based on using intrinsic coordinates by estimating the Frenet frames along the curve at each centreline point, using the central difference approximation of the derivative among the interior points and forward/backward differences at the ends. As the geometry of the centreline trajectory is known, we can, therefore, calculate the directions of the tangent (*T*), normal (*N*), and binormal vectors (*B*), and we can use them to build a tube-like structure in three dimensions.

At each centreline point *A* along the centreline, we need to build a 3D circle. Assuming *X* = (*x*, *y*, *z*) are the coordinates of the centreline point *A*, the 3D coordinate of the circle becomes

(7) $$\begin{array}{c} X=X+r\cdot \left(\;N\cdot \cos\theta +B\cdot \sin\theta \;\right), \end{array}$$

where *θ* is varied between 0 and 360° at intervals *L* = 2*π*/*s* − 1, *s* being the number of subdivisions.

## 3. Results

**Figure 5:**
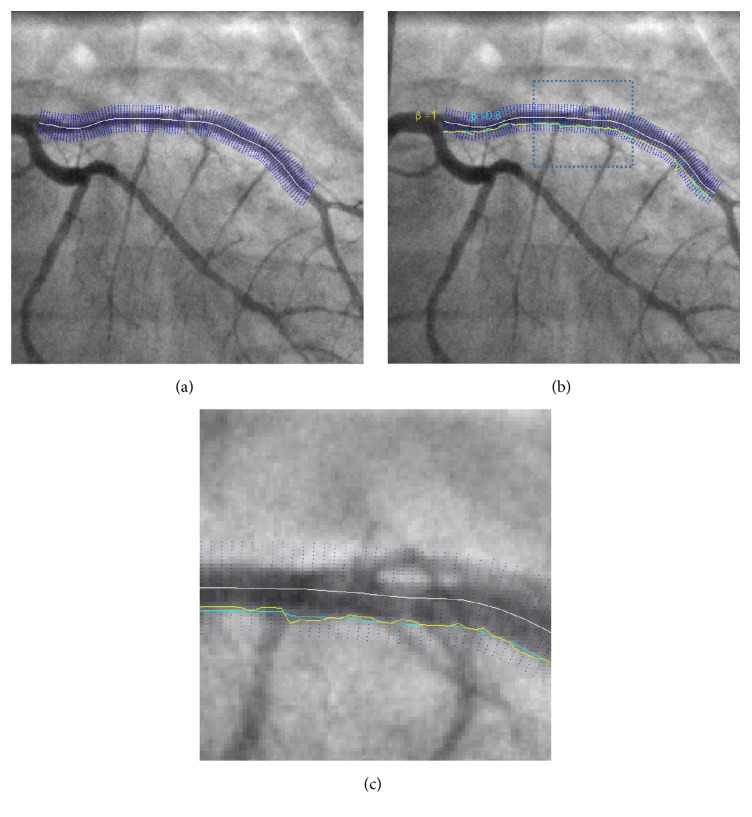
Effect of the *β* parameter on the smoothness of the final selected vessel: (a) manually drawn centreline shown (white), alongside the normals (blue), (b) extracted wall boundaries using *β* = 1 (yellow), and *β* = 0.8 (cyan), (c) zoom-in version of rectangular region specified in (b).

**Figure 6:**
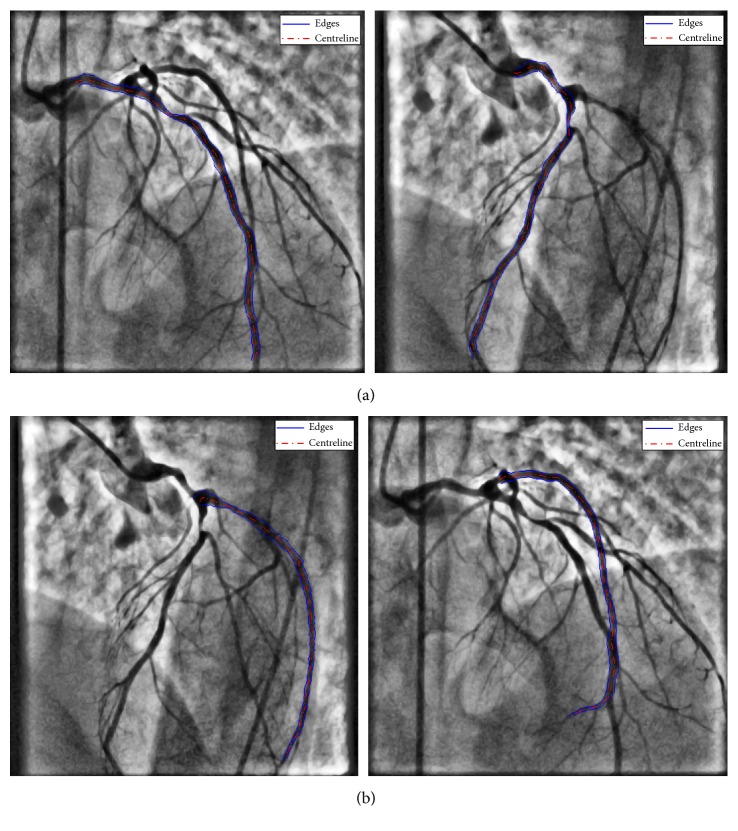
Vessel edge extraction using the proposed dynamic programming method: (a) LAD edge extraction 1st and 2nd projections, left and right, respectively, and (b) LCX vessel edge extraction 1st and 2nd projections, left and right, respectively.

Simulations were run under Matlab R2010a using an Intel Xeon 5130, 2.00 GHZ processor with 8 GB of RAM. Edge detection was performed on all the branches selected in the centreline extraction stage. Based on the results of multiple simulations, the hyperparameter *β* was chosen to be 0.75 to place more weight on the edge magnitudes. In order to better illustrate the effect of the weighting factor *β*, the effect of varying this is shown in Figure 5 for two different values near the centreline of the vessel. The weighting factor *β* can be varied between 0 and 1. A weight of one corresponds to the smoothest path, whereas a weight of zero forces the path through the brightest pixels (highest edge magnitude) in the edge image. Simulation times take on average 0.57 seconds to complete including the LAD, LCX vessels, and three subbranches. The results of the vessel edge extraction illustrate the robustness of the approach and how branching points do not alter the shape of the LAD/LCX arteries, as shown in Figure 6.

In order to validate the accuracy of the edge extraction results, the results were compared to ground truth segmentations. However, obtaining ground truth results is not an easy task and has been known to be a hard problem in image analysis and pattern recognition systems [36]. The latter is due to the implicitly subjective nature of image labelling by human experts. The common approach in vessel segmentation has often been to approximate ground truth by the creation of a human expert-generated manual segmentation also known as “gold standard” to which computer-generated segmentations can be evaluated. Thus, it seems reasonable that a more robust approach to the creation of a gold standard is to combine multiple human generated manual segmentations or choose the best one among them. Therefore, given a set of manual segmentations generated by multiple experts, we wish to obtain a single binary segmentation, which will be considered the gold standard.

A solution is to use redundancy not only for identifying the correct vessel annotations for each angiogram image but also for evaluating the labelling quality of the experts. We use the method proposed in [37], where the authors use a Maximum likelihood (ML) approach to ground truth generation.

**Figure 7:**
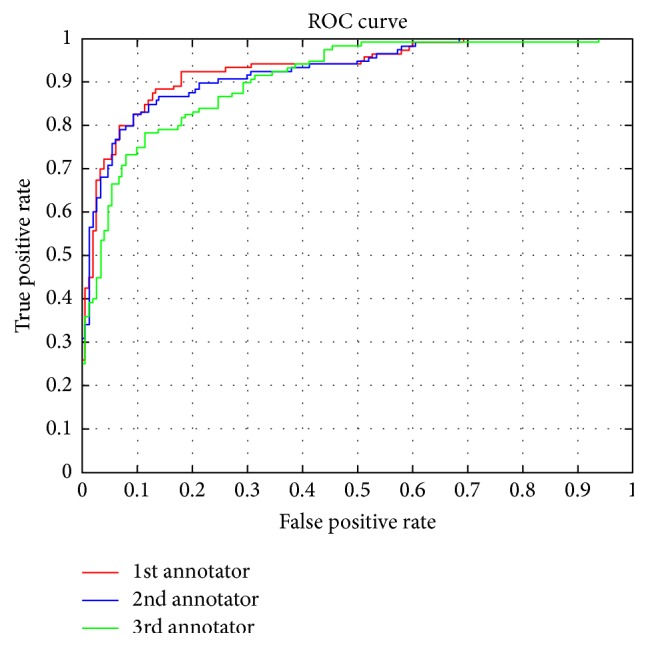
ROC comparison for the three expert annotators.

When using the latter method, we iterate the algorithm until convergence, following two steps: (1) estimate the correct label for each vessel branch on each projection, using labels assigned by three radiologists (1 for edge and 0 for nonedge), and (2) estimate the quality of the radiologists' labelling capabilities by comparing the submitted annotations to the inferred correct answers. The final output is a set of (estimated) correct vessel edges for each branch on every projection and a confusion matrix for each radiologist, listing the error probabilities for each of them. From the confusion matrix, we can directly measure the overall error rate for each labeller as the sum of the nondiagonal elements of the confusion matrix (appropriately weighted by priors). This results in a single, scalar value as the quality score for each radiologist. In our studies, the first radiologist turned out to provide the most accurate annotations. The ROC generated from the described algorithm is shown in Figure 7.

It is easy to verify that the first curve stretching almost into the top left corner represents the performance of the superior model (for a TPR = 0.9, the method commits virtually no false positives). Next, having obtained the best labeller, its corresponding labelled edge curves need to be compared with the extracted curves using the proposed method. In order to compare the distance between the vessel edge extraction results to that of the 1st annotator (i.e., ground truth), we use the Hausdorff distance between the curves to evaluate the difference. The Hausdorff distance is defined as

(8) δHx,y=max⁡max⁡min⁡dx,y,max⁡min⁡dx,yx∈X  y∈Y,

where *d* is the underlying metric in the plane, in this case the Euclidian distance, and*X* and *Y* are the two sets of points describing the two curves to be compared. The results of the average distance for the extracted edge curves to the ground truth curve are shown in Table 1.

As it can be observed from Table 1, there is little discrepancy between the ground truth curves and the extracted vessel walls obtained using the proposed algorithm. One of the advantages of the proposed method is its insensitivity to branching at bifurcations, which further adds to the robustness of the algorithm.

**Figure 8:**
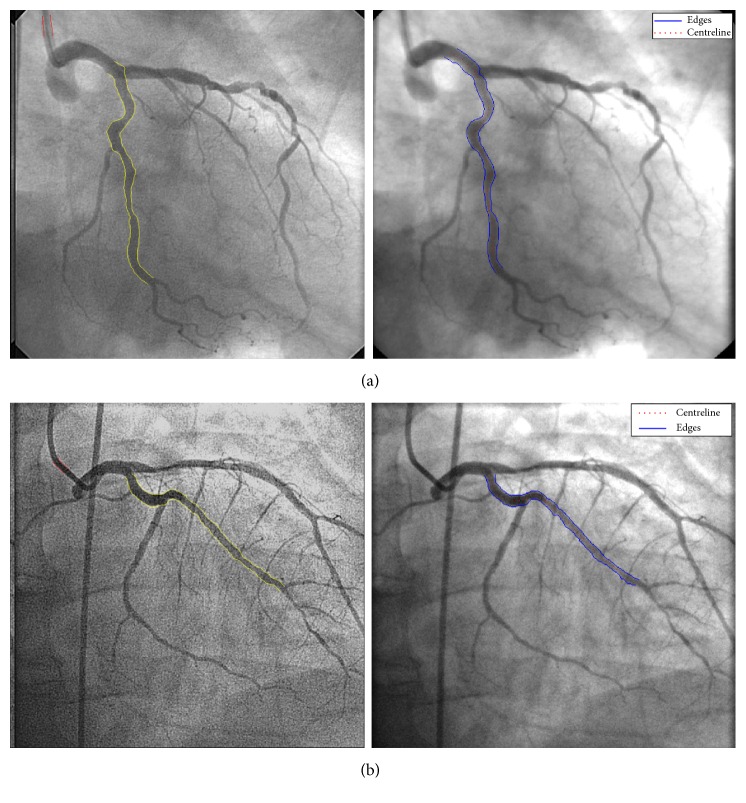
Vessel edge extraction comparison using the proposed dynamic programming method and QCA-CMS 6.0, left and right, respectively: (a) LAD, (b) LCX.

In order to have another comparison to an existing state-of-the-art vessel wall delineation method, we compared the proposed method to the automated vessel contour detection with manual correction methodology performed with QCA-CMS version 6.0 (Medis, Leiden, The Netherlands). The calibration procedure is initiated by the user defining start and end points in the catheter segment, which are connected using the Wavepath algorithm [38, 39]. The catheter segment chosen may be straight or curved, but it must not taper. The actual contour detection procedure is carried out in two iterations. In the first iteration, the contours are detected by defining scanlines perpendicular to the detected pathline and calculating for each point along the scanline the edge strength on the basis of the first- and second-order derivative functions. This process is repeated for each point along the scanline in increments of approximately 0.1 mm. The contours are then detected using the minimal cost analysis (MCA) algorithm [40]. In the second iteration, scanlines are defined perpendicularly to each of the individual right and left contours and the contour detection process is repeated. During this second iteration, a priori information about the catheter segment being a cylindrical structure characterized by parallel, although not necessarily straight, boundaries is taken into account in the edge detection process. In addition, the edge detection algorithm is modified by data from the Modulation Transfer Function (MTF) of the entire imaging system to correct for its limited resolution. This process results in two contours which are parallel to each other but may still be curved. After the boundaries of the catheter have been detected within an image, the average diameter of the catheter segment, expressed in pixels, is calculated.  Figure 8 shows a comparison between the proposed methodology and the QCA-CMS. The average Hausdorff distances for the extracted edge curves using QCA-CMS and the proposed method are shown in Table 2.

Having obtained the 2D projected vessel wall point coordinates (i.e., edge curves), the next step is to reconstruct the vessel walls in three dimensions. As we are only using two image projections, we assume a circular vessel cross section at each centreline point. In order to build the vessel surface in 3D, we proceed as follows. First, we use the 3D centreline reconstruction algorithm [33] to project the extracted vessel wall curves onto 3D space. Next, at each centreline point, we calculate the Euclidean distance of that particular centreline point to its corresponding edge projected curve points along its normal. We use the average of the distances as the radius for building tilted 3D circles in the next stage. Once we obtained the radii of the vessel at each centreline point, we build a Frenet frame moving from the start point toward the end point on each vessel, at each centreline point. Next, we generate a 3D circle using the binormal and normal vectors, as described in the previous section. The resulting circles are meshed, according to the desired subdivision to yield the projections. In order to obtain the final arterial tree, we carry out the same procedure for all other vessels and branches selected in order to obtain the final full three-dimensional reconstruction. The result of the 3D reconstruction for the selected branches selected of Figure 2(a) are displayed in Figure 3(b). The 3D vessel not only allows better quantification of vessel information but also paves the way for subsequent tessellation to carry out CDF analysis. The developed GUI allows the final 3D mesh to be exported as “stl” or “obj” file. Moreover, other relevant pieces of information such as torsion and curvature are obtained as outputs for additional information describing vessel morphology.

## 4. Conclusions

In this research, we introduced a new edge detection method for accurate reconstruction of coronary vessel walls in three dimensions from two monoplane angiogram images. The multistage approach offers a new automatic paradigm to reconstruct angiogram images from 2D projections. One of the main features of the proposed method is the novel robust edge extraction algorithm for the extraction of vessel edges using dynamic programming. The latter method is insensitive to the presence of bifurcation and branching points, which is a major problem in coronary vessels edge extraction. The user clicks on the desired segments of vessels and the process thereafter is fully automatic requiring no user interaction. The results show the robustness of the approach and its potential use in coronary image-guided interventions.

We introduced a pipeline for generation of patient-specific 3D models of coronary vessels using anatomical information from angiograms. Further work is in progress on the Finite Element (FEM) mesh models of the resulting reconstructions by adding boundary and material properties, with the ultimate goal of creating realistic vessel models. The resulting vessel models could be further coupled with the method of [35], incorporating blood flow in a virtual environment to carry out hemodynamic analysis of coronary blood flows in patient coronary arteries.

## Figures and Tables

**Table 1:** Discrepancies between ground truth and extracted vessel walls.

Patient	Average Hausdorff distance to ground truth			
	LAD 1st proj.	LAD 2nd proj.	LCX 1st proj.	LCX 2nd proj.
1	0.0039	0.0041	0.0035	0.0037
2	0.0032	0.0039	0.0031	0.0031
3	0.0041	0.0038	0.0039	0.0042
4	0.0029	0.0031	0.0029	0.0029
5	0.0034	0.0037	0.0033	0.0035

**Table 2:** Discrepancies between the proposed method and QCA.

Patient	Average Hausdorff distance QCA and the proposed method			
	LAD 1st proj.	LAD 2nd proj.	LCX 1st proj.	LCX 2nd proj.
1	0.0068	0.0043	0.0052	0.0054
2	0.0051	0.0035	0.0069	0.0057
3	0.0059	0.0045	0.0050	0.0053
4	0.0034	0.0037	0.0035	0.0037
5	0.0062	0.0047	0.0063	0.0047
